# Supplementary material for: Proteome-scale characterisation of motif-based interactome rewiring by disease mutations
Source: Mol Syst Biol. 2024 Jul 15;20(9):1025–48. doi: 10.1038/s44320-024-00055-4 (PMC11369174; doi:10.1038/s44320-024-00055-4)
Supplement: Supplementary file 1 — Appendix [file 44320_2024_55_MOESM1_ESM.pdf]

Proteome-scale characterisation of motif-based interactome rewiring by disease mutations

Johanna Kliche[1], Leandro Simonetti[1], Izabella Krystkowiak[2], Hanna Kuss[1,3], Marcel Diallo[4], Emma Rask[1], Jakob Nilsson[4], Norman E. Davey[2,#] and Ylva Ivarsson[1,#]

[1] Department of Chemistry - BMC, Box 576, Husargatan 3, 751 23 Uppsala, Sweden.

[2] Division of Cancer Biology, Institute of Cancer Research, Chester Beatty Laboratories, 237 Fulham Road, SW3 6JB, Chelsea, London, UK.

[3] Current address: University of Münster, Institute of Pharmaceutical and Medicinal Chemistry, DE-48149 Münster, Germany

[4] Novo Nordisk Foundation Center for Protein Research, Faculty of Health and Medical Sciences, University of Copenhagen, Copenhagen, Denmark.

Table of contents: Appendix figures

Appendix Figure S1. Analysis of the GenVar_HD2 library composition………………. Page 2

Appendix Figure S2. Comparison of the distribution of the p-values of the most significant shared GO terms for domain (bait) - mutation………………………………………………… Page 3

Appendix Figure S3. V-shaped plot of the domain-mutation pairs……………………. Page 3

Appendix Figure S4. FP-monitored saturation binding experiments…………………. Page 4

Appendix Figure S5. FP-monitored displacement experiments………………………. Page 5

Appendix Figure S6. ITC curves of MAP1LC3B with the $BRCA2_{292-307}$ peptide……. Page 6

Appendix Figure S7. Co-immunoprecipitation experiments………………………….... Page 7

Appendix Figure S8. Representative confocal microscopy images of wild-type EGFP-ABRAXAS1……………………………………………………………………………………… Page 8

Appendix Figure S9. Representative confocal microscopy images of R361Q EGFP-ABRAXAS1.…………………………………………………………………………….… Page 9

Appendix Figure S10. Representative confocal microscopy images of wild-type EGFP-CDC45.……………………………………………………………………………………. Page 10

Appendix Figure S11. Representative confocal microscopy images of R157C EGFP-CDC45.……………………………………………………………………………………. Page 11

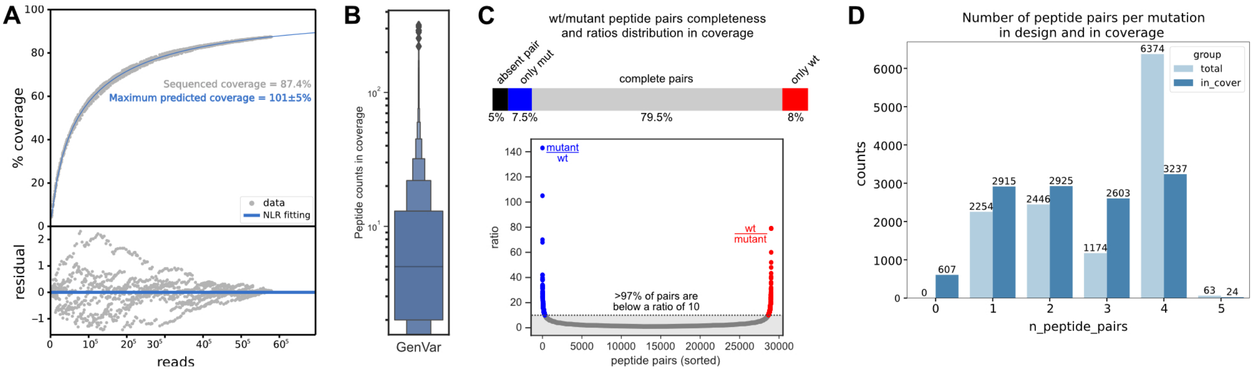

Appendix Figure S1. Analysis of the GenVar_HD2 library composition. A. Observed (grey) and predicted (blue) peptide coverage of the GenVar_HD2 library after sequencing unchallenged *naive* aliquots (top). The residuals of the non-linear regression fitting are shown at the bottom. **B.** NGS count distribution associated with peptides in the library. **C.** Top: GenVar_HD2 library wild-type (wt)/mutant (mut) pairs completeness in the observed coverage. Bottom: Ration between NGD counts for wt and mutant paris. **D.** Number of overlapping wt/mut peptide pairs covering each mutation in the GenVar_HD2 library design (light blue) and in the observed coverage (blue).

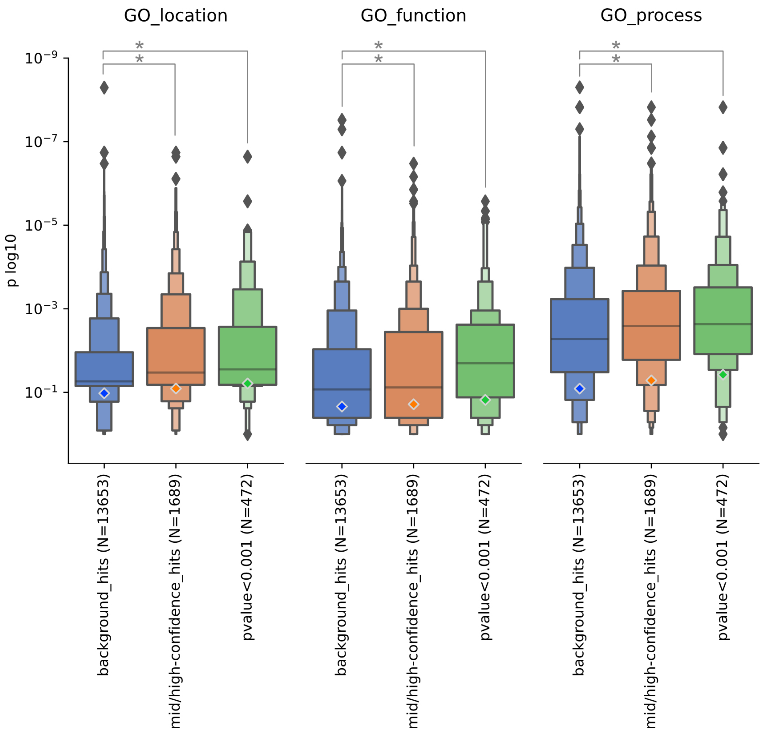

Appendix Figure S2. Comparison of the distribution of the p-values of the most significant shared GO terms for domain (bait) - mutation (prey) pairs (**Dataset EV4**). The background (blue) group corresponds to all domain-mutation selection results with confidence levels of 0 or 1, while the mid/high-confidence (orange) group includes all domain-mutation pairs with confidence scores of 2 or more. The subset of mid/high-confidence domain-mutation pairs where the mutation enhances/diminishes the interaction (that is with a p-value < 0.001, **Figure 2B**) is shown in green. Asterisk (*) denotes Mann-Whitney test p-value lower than 0.001 for mean comparison of the depicted groups.

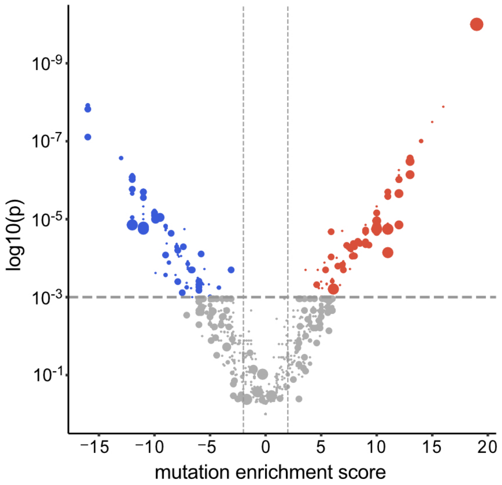

Appendix Figure S3. V-shaped plot of the domain-mutation pairs. Shown are pairs for which at least one individual peptide pair was found to indicated significant binding preferences for wild-type or mutant peptide. The size of the circle encodes the number of the significant peptide pairs behind the domain-mutation pair.

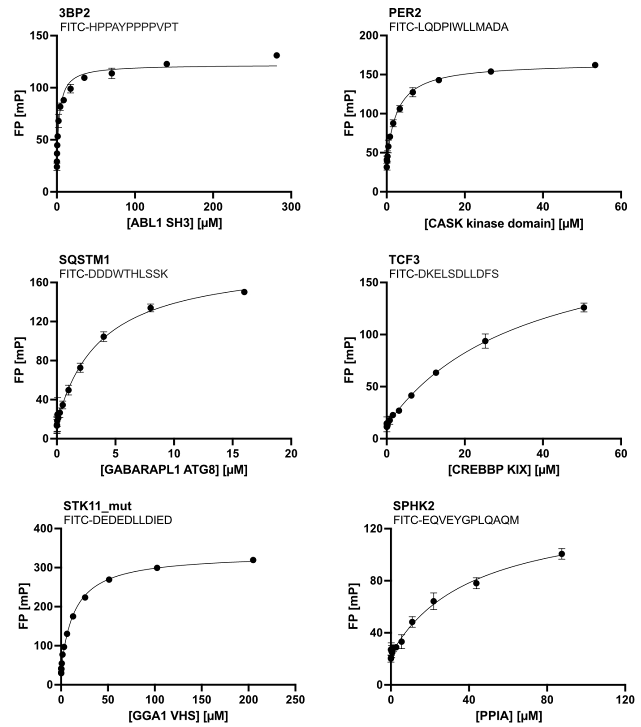

Appendix Figure S4. FP-monitored saturation binding experiments. Depicted is the binding of ABL1 SH3, CASK kinase domain, CREBBP KIX, GABARAPL1 ATG8, GGA1 VHS domain and PPIA to their respective FITC-labelled peptides. Measurements were in technical triplets.

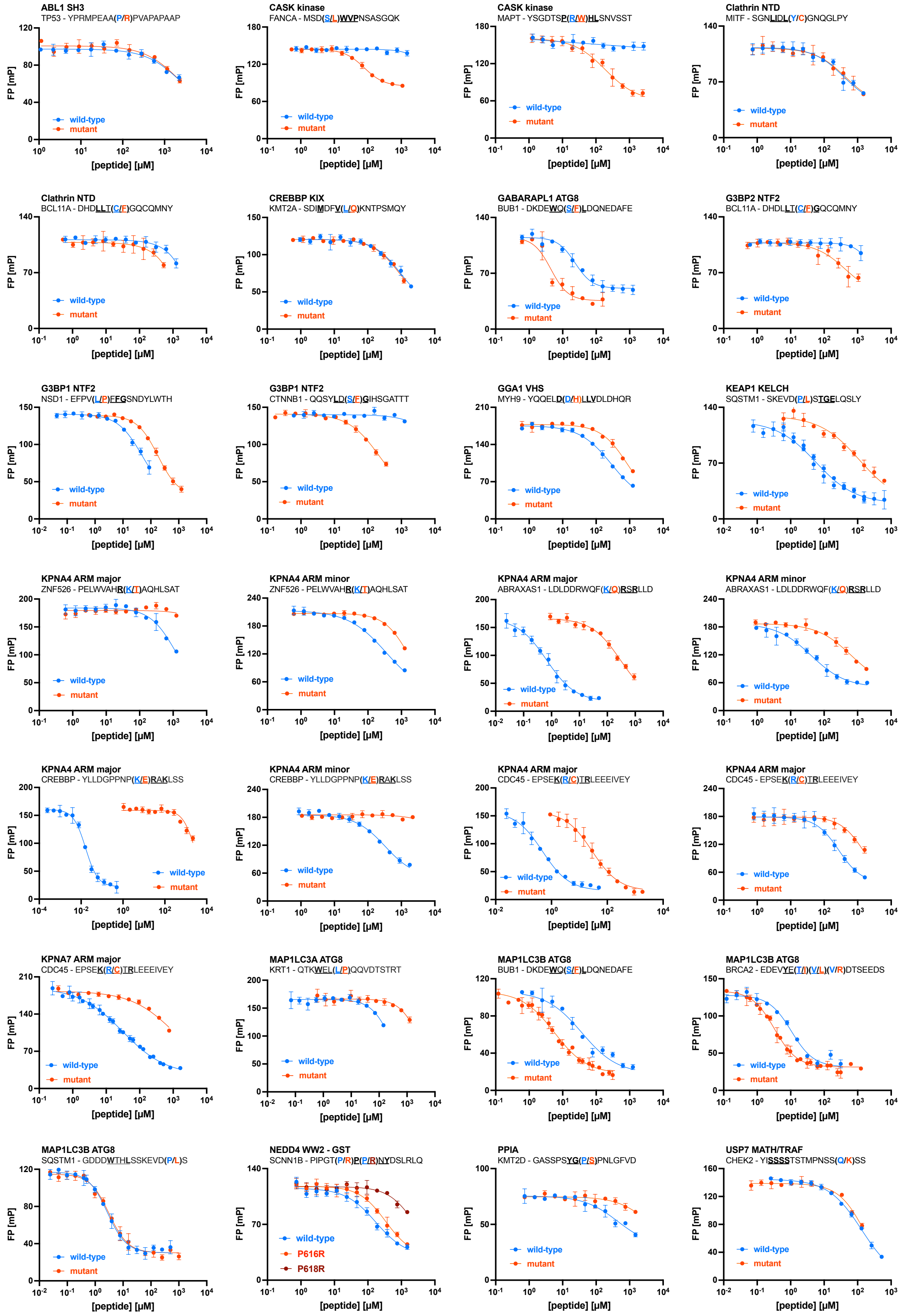

Appendix Figure S5. FP-monitored displacement experiments. Specified are the protein domain used and the wild-type/mutant displacing peptide. Measurements were in at least technical triplets.

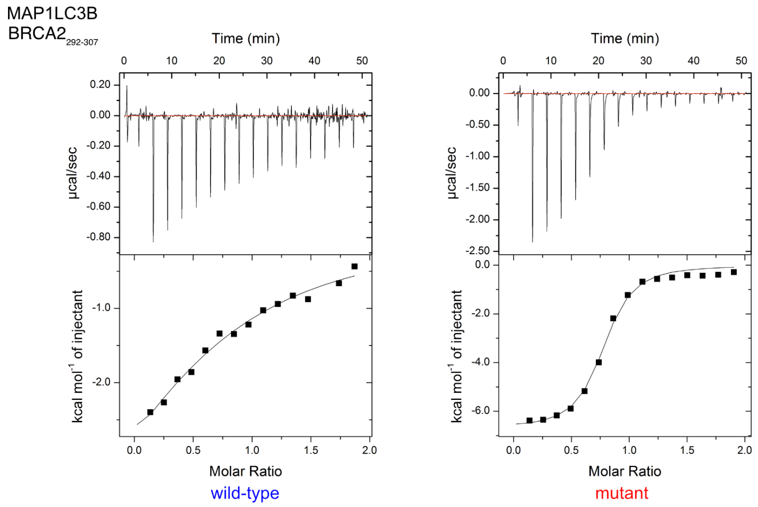

Appendix Figure S6. ITC curves of MAP1LC3B with the BRCA2$_{292\text{-}307}$ peptide. Measurements were in technical duplicates for the wild-type and technical triplets for the mutant peptide.

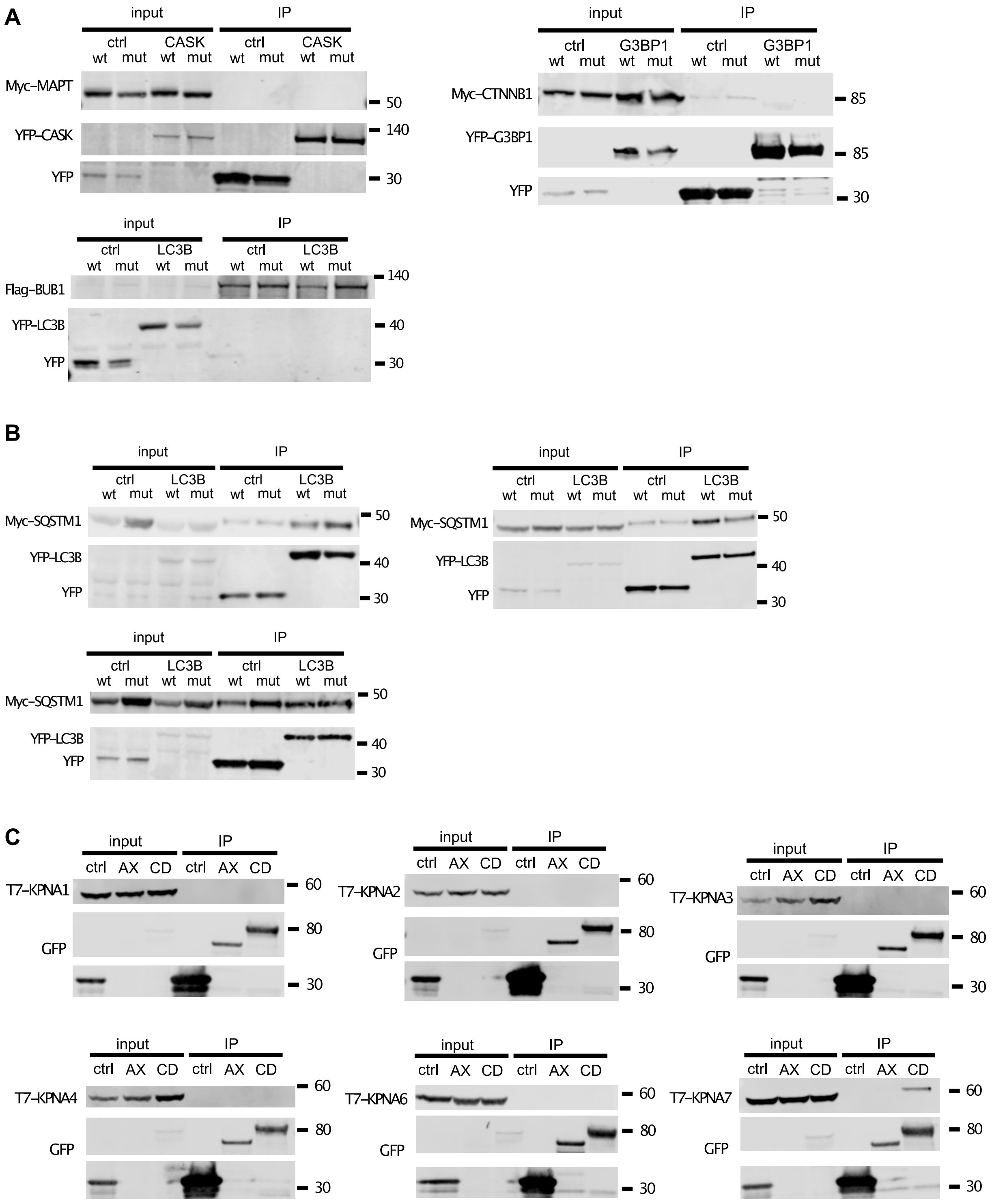

Appendix Figure S7. Co-immunoprecipitation experiments. A: YFP-tagged CASK and Myc-tagged wild-type/ R348W MAPT (TAU-D isoform), YFP-G3BP1 and Myc-tagged wild-type/ S33F CTNNB1, as well as YFP-MAP1LC3B and Flag-tagged wild-type/ S492F BUB1. B: YFP-tagged MAP1LC3B and Myc-tagged wild-type/ P348L SQSTM1. Blots from three biological replicates. C: EGFP-tagged wild-type ABRAXAS1 (AX) or CDC45 (CD) with T7-tagged KPNA1, 2, 3, 4, 6 and 7. A single repeat was performed for the negative co-immunoprecipitations, whereas the positive interactions between CDC45 and KPNA7 was probed in three biological replicates.

ABRAXAS1 wild-type

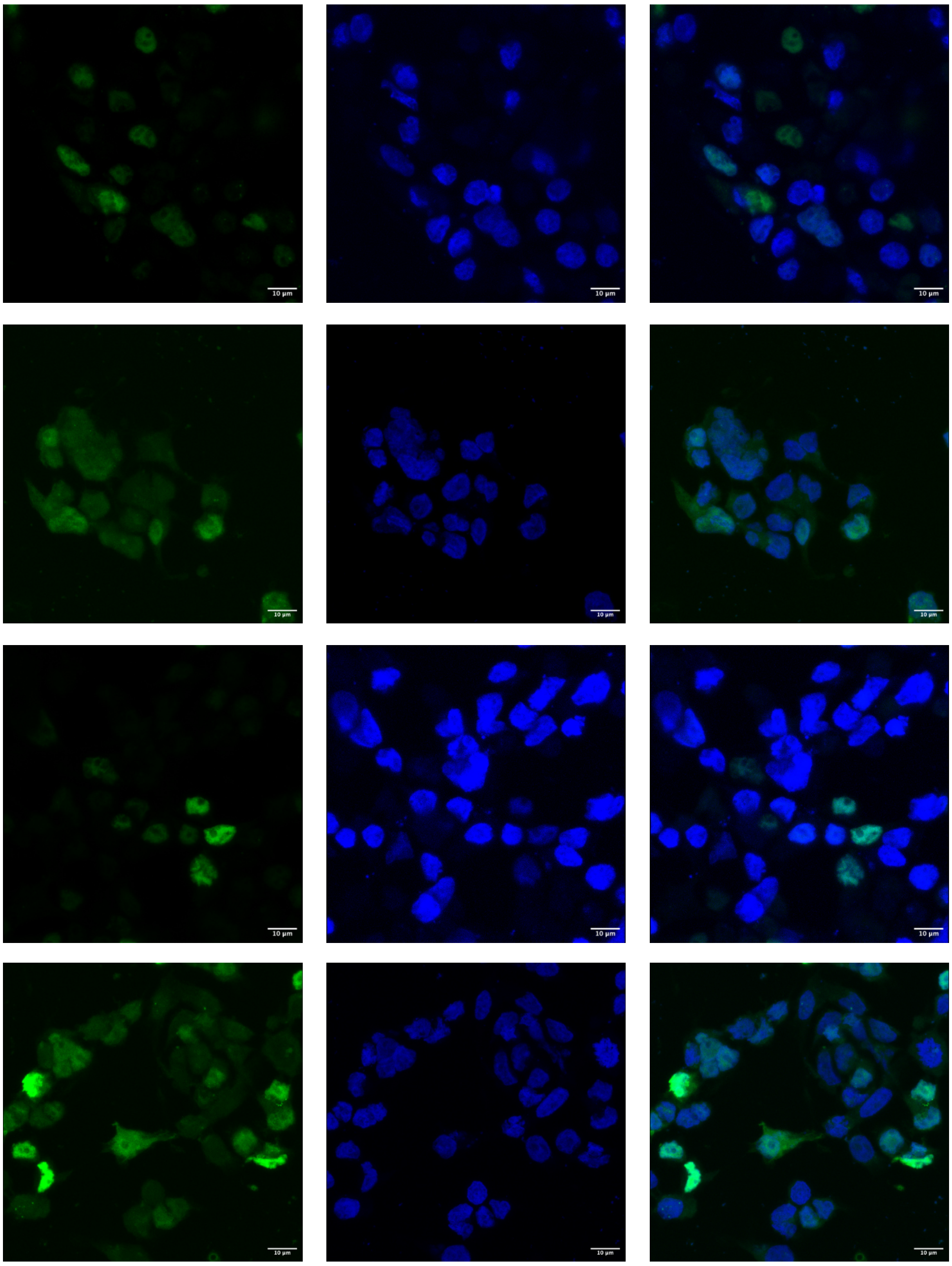

Appendix Figure S8. Representative confocal microscopy images of wild-type EGFP-ABRAXAS1. Experiments were performed in biological triplets. Data from this figure is also represented in Figure 5H.

ABRAXAS1 R361Q

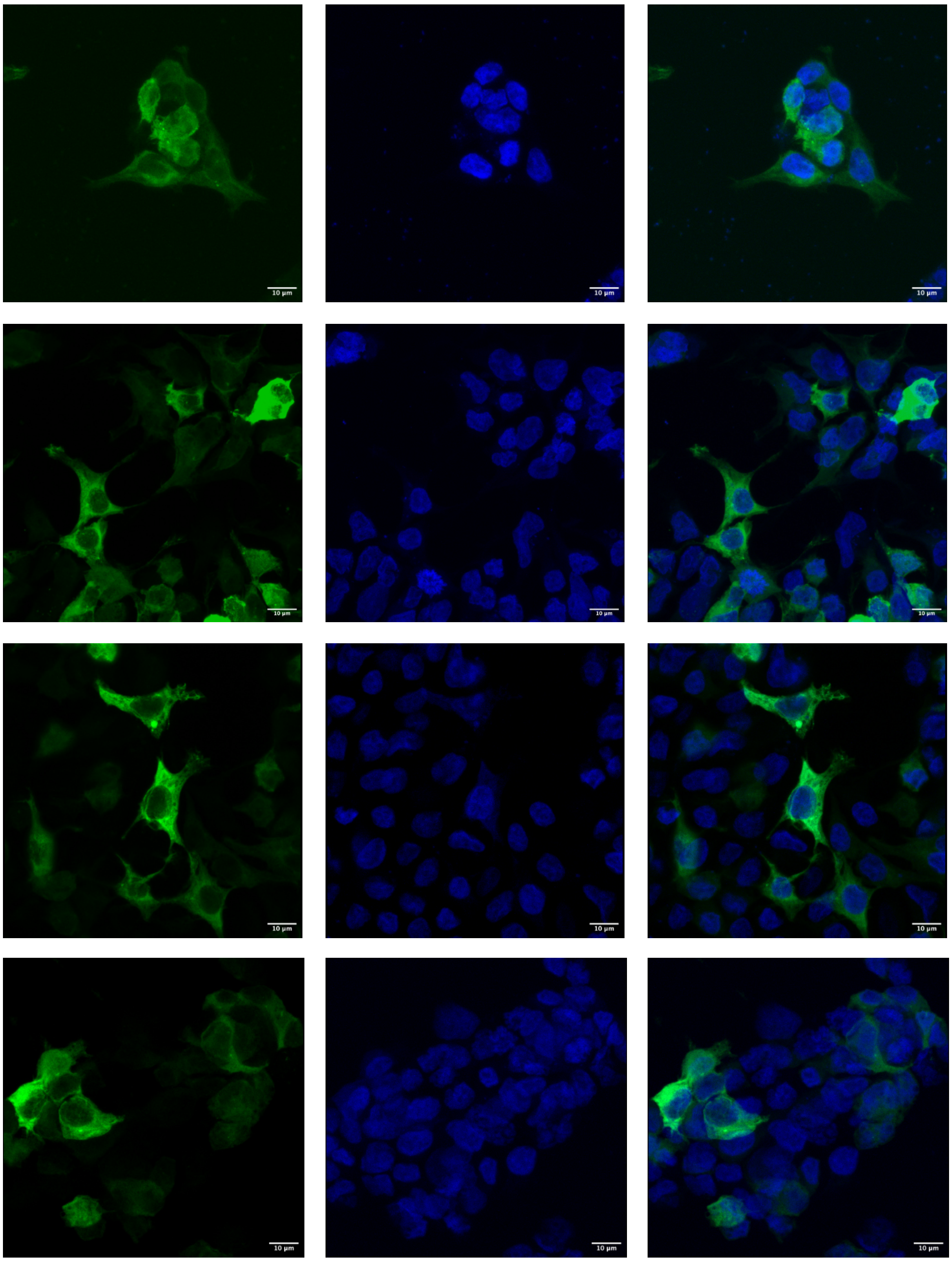

Appendix Figure S9. Representative confocal microscopy images of R361Q EGFP-ABRAXAS1. Experiments were performed in biological triplets.

CDC45 wild-type

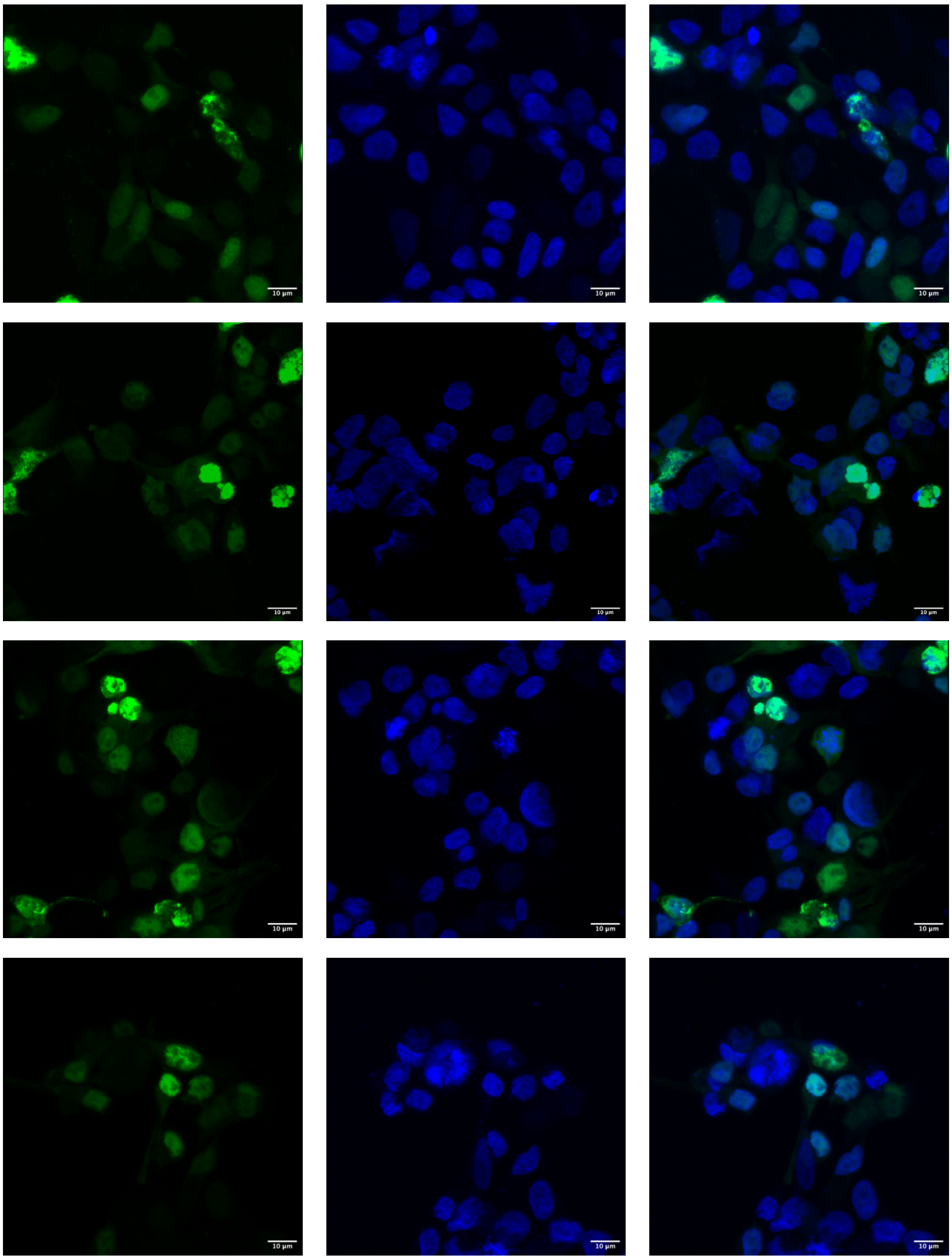

Appendix Figure S10. Representative confocal microscopy images of wild-type EGFP-CDC45. Experiments were performed in biological triplets.

CDC45 R157C

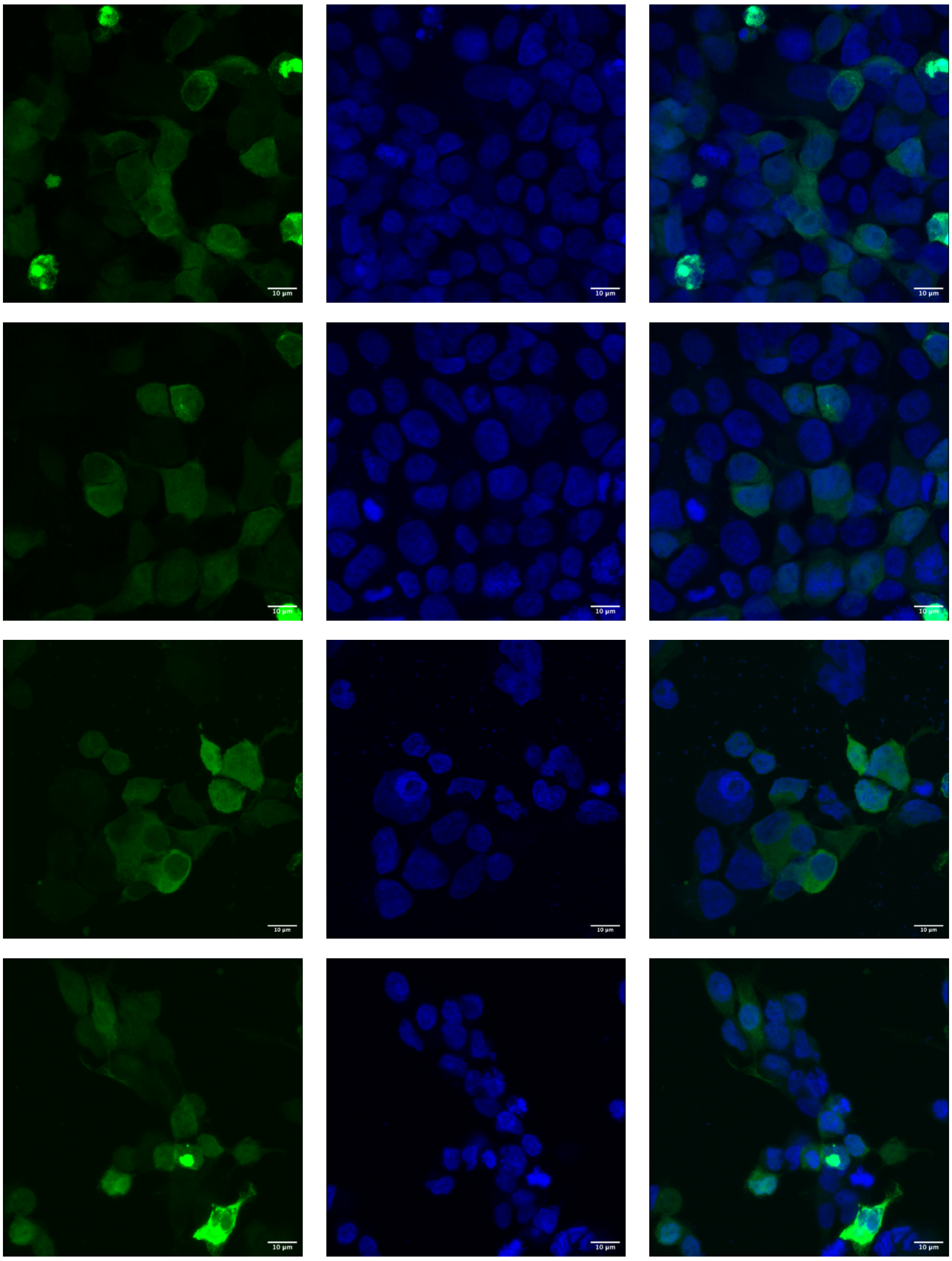

Appendix Figure S11. Representative confocal microscopy images of R157C EGFP-CDC45. Experiments were performed in biological triplets.
